# Progesterone Receptor Coregulators as Factors Supporting the Function of the Corpus Luteum in Cows

**DOI:** 10.3390/genes11080923

**Published:** 2020-08-12

**Authors:** Robert Rekawiecki, Karolina Dobrzyn, Jan Kotwica, Magdalena K. Kowalik

**Affiliations:** Institute of Animal Reproduction and Food Research of the Polish Academy of Sciences, Tuwima 10, 10–747 Olsztyn, Poland; k.dobrzyn@pan.olsztyn.pl (K.D.); j.kotwica@pan.olsztyn.pl (J.K.); m.kowalik@pan.olsztyn.pl (M.K.K.)

**Keywords:** progesterone receptor coregulators, P300, CREB, SRC-1, NCOR-2, corpus luteum, cow

## Abstract

Progesterone receptor (PGR) for its action required connection of the coregulatory proteins, including coactivators and corepressors. The former group exhibits a histone acetyltransferase (HAT) activity, while the latter cooperates with histone deacetylase (HDAC). Regulations of the coregulators mRNA and protein and HAT and HDAC activity can have an indirect effect on the PGR function and thus progesterone (P4) action on target cells. The highest mRNA expression levels for the coactivators—histone acetyltransferase p300 (*P300*), cAMP response element-binding protein (*CREB*), and steroid receptor coactivator-1 (*SRC-1*)—and nuclear receptor corepressor-2 (*NCOR-2*) were found in the corpus luteum (CL) on days 6 to 16 of the estrous cycle. The CREB protein level was higher on days 2–10, whereas SRC-1 and NCOR-2 were higher on days 2–5. The activity of HAT and HDAC was higher on days 6–10 of the estrous cycle. All of the coregulators were localized in the nuclei of small and large luteal cells. The mRNA and protein expression levels of the examined coactivators and corepressor changed with the P4 level. Thus, P4 may regulate CL function via the expression of coregulators, which probably affects the activity of the PGR.

## 1. Introduction

Progesterone (P4) is a steroid hormone produced by the follicle, corpus luteum (CL), and placenta; it plays a crucial role in regulating the length of the estrous cycle, blastocyst implantation, and the maintenance of pregnancy in many species of mammals, including cows [1]. The genomic action of P4 is mediated by their specific nuclear receptors (PGRs). These receptors are ligand-regulated transcription factors activated by P4. The cellular response to P4 is mediated by two isoforms of the PGR: isoform A (PGRA) and isoform B (PGRB). Both isoforms are transcribed from the same gene under the influence of two different promoters [2]. They differ in their length—PGRB is longer than PGRA by approximately 164 nucleotides in humans; in other species, this difference is 128–165 amino acids [2]. Each isoform performs a different action. PGRB acts as a potent activator of genes that are dependent on P4, whereas PGRA is a weak activator of such genes [3].

The inactive form of the PGR receptor is present in the cytosol and connected to the heat shock protein (HSP) [4]. After the hormone binds to the receptor, these proteins dissociate, and PGR translocates to the cell nucleus. Here, it dimerizes and connects to the promoter region of the gene expressed. The last step in PGR activation is the connection of the receptor’s additional elements, which are called coregulators and modulate receptor action. This group of proteins interacts with the receptor complex without binding to the DNA of the target gene sequence [5]. Coregulators are divided into two groups of proteins: coactivators (proteins that enhance the transcription of target genes) and corepressors (proteins that inhibit the transcription of such genes) [6]. Coactivators have internal histone acetyltransferase (HAT) activity. By acetylating histone proteins, HAT loosens chromatin, which results in an increased expression of various genes [7]. In contrast, corepressors interact with histone deacetylases (HDACs), which deacetylate histone proteins, thereby increasing chromatin condensation and inhibiting the transcription of the target genes [8].

Both coactivators and corepressors may exhibit variable expression in the estrous cycle, as shown in the human endometrium [9], as well as the bovine endometrium and CL [10]. Therefore, changes in the mRNA and protein levels of coregulators, as well as HAT and HDAC activity, can affect the PGR function of the CL and the uterus, thereby regulating the action of P4 on target cells. Therefore, this study aims to evaluate cellular distribution and the levels of mRNA expression and protein for the coactivators—histone acetyltransferase p300 (P300), cAMP response element-binding protein (CREB), and steroid receptor coactivator-1 (SRC-1)—and the corepressor, nuclear receptor corepressor-2 (NCOR-2), in luteal cells during the estrous cycle in cows. We also determine the activity of HAT and HDAC in the CL during the estrous cycle.

## 2. Materials and Methods

### 2.1. Tissue Collection

Corpora lutea from non-gravid cows and mature heifers were harvested from a commercial slaughterhouse within 20 min of death. Tissues from the four stages of the estrous cycle (days 2–5, 6–10, 11–16, and 17–20) were frozen in liquid nitrogen, transported to the laboratory, and stored at −80 °C until further use. The days of the estrous cycle were determined by criteria reported by Ireland [11]. The CLs were transported to the laboratory in ice-cold phosphate-buffered saline (PBS) within 1 hour of death for the isolation of nuclear proteins for determination of HAT and HDAC activity. Corpus luteum sections for immunohistochemistry were fixed with 4% paraformaldehyde in 0.1 M PBS (pH 7.4) for 24 h and washed with distilled water, followed by dehydration in an ethanol gradient and embedding in paraffin. The deeply frozen tissues were homogenized with a Retsch MM–2 vibratory mill (Retsch GmbH, Haan, Germany). Individual portions of tissue powder were separated for the isolation of RNA and protein level determination. Nuclear proteins were isolated using a Nuclear/Cytosol Fractionation Kit (Biovision, Milpitas, CA, USA), according to the manufacturer’s instructions, and stored at −80 °C until further analysis.

### 2.2. Progesterone Determination

We determined P4 concentrations by enzyme immunoassay (EIA), as previously reported by Prakash [12], using a reader plate (Multiscan EX, Labsystem, Helsinki, Finland) for the measurement at an absorbance of 450 nm. Progesterone was extracted from CL tissue using petroleum ether [13]. Recovery of this hormone averaged 90%; data were corrected for procedural losses. Progesterone labeled with horseradish peroxidase was used at a final dilution of 1:40,000. P4 antiserum (IFP4) was used at a final dilution of 1:60,000 and has been previously characterized by Kotwica [14]. The range of the standard curve was 0.1–25 ng/mL, and the sensitivity of the procedure was 0.15 ng/mL. The values of steroids shown on the graph were calculated per gram of CL tissue. Intra- and inter-assay coefficients of variation for the control samples were 6.2% and 7.7% on average, respectively. The relationship between the added and measured amounts of hormone (*n* = 5) was significant (*r* = 0.96).

### 2.3. Immunohistochemistry

For immunohistochemical localization, CL sections of 6 μm thickness from days 6–10 and 17–20 were cut from paraffin-embedded samples and mounted on Super Frost Plus microscope slides. The sections were deparaffinized in xylene and rehydrated in a series of ethanol dilutions (100%, 96%, 70%, H2O). Antigen retrieval was performed by pressure cooking in 0.01 M sodium citrate with 0.05% Tween 20 (pH 6.0). After blocking the endogenous peroxidase activity with BLOXALL Endogenous Peroxidase and Alkaline Phosphatase Blocking Solution (Vector Laboratories, Peterborough, UK) for 15 min, we incubated the sections with 2.5% normal horse serum (NHS) to block nonspecific binding sites (45 min at room temperature). Next, the sections were incubated overnight at 4 °C with primary antibodies for coactivators: P300 (1:70) (Cohesion Biosciences, London, UK), CREB (1:400) (Cohesion Biosciences, London, UK), SRC-1 (1:35) (Sigma, Poznan, Poland), and corepressor NCOR–2 (1:50) (Sigma, Poznan, Poland). For the negative control sections, primary antibodies were replaced with 2.5% normal horse serum (NHS). On the next day, the sections were washed in Tris-buffered saline (TBS) three times for 10 minutes and incubated with the ImmPRESS Reagent (Vector Laboratories, Peterborough, UK) at 4 °C for 30 min (this reagent contains the second antibody). After washing three times for 10 min in TBS, the sections were visualized with 3,3′-diaminobenzidine (DAB; Vector Laboratories, Peterborough, UK). Next, they were rinsed, counterstained with Mayer’s hematoxylin (1 min), dehydrated, covered with mounting medium (DPX; POCh, Gliwice, Poland), and then examined using a Zeiss Axio Imager Z1 microscope (Zeiss, Oberkochen, Germany).

### 2.4. RNA Isolation and Reverse Transcription

Total RNA was isolated from homogenized tissue using a Universal RNA Purification Kit (EURx, Gdansk, Poland) according to the manufacturer’s instructions, based on the method described previously by Chomczynski and Sacchi [15]. Isolated RNA was stored at −80 °C until further analysis. The concentration and purity of the RNA were determined by measuring the absorbance at 260 nm and 280 nm wavelengths using a NanoDrop 1000 spectrophotometer (Thermo Scientific, Wilmington, DE, USA). DNase–treated RNA (1 μg) was reverse transcribed using the TRANSCRIPTME cDNA Synthesis Kit (Blirt, Gdansk, Poland) according to the manufacturer’s instructions.

### 2.5. Real-Time PCR

Real-Time PCR was performed by means of the Applied Biosystems 7900 Real–time PCR System (Applied Biosystems, Foster City, CA, USA) using the Power SYBR Green PCR Master Mix (Applied Biosystems, Foster City, CA, USA). Table 1 shows the oligonucleotide primers used for PCR amplification and the expected product sizes of coactivators P300, CREB, and SRC-1, corepressor NCOR-2, and the housekeeping gene TATA-Box Binding Protein (TBP). The real-time PCR reaction mixture (20 μL) consisted of 100 ng cDNA, 10 μL Master Mix, and 0.2 mM PCR primers for each gene of interest. The PCR protocol had an initial denaturation step (10 min at 95 °C), followed by 40 cycles of denaturation (15 s at 95 °C) and annealing and extension (1 min at 60 °C). All reactions (*n* = 5) were performed in duplicate.

### 2.6. Western Blot

We prepared the protein samples using radioimmunoprecipitation assay buffer (RIPA) with protease inhibitors (25 mM Tris–HCl, pH 7.6; 150 mM NaCl, 1% Triton X–100, 1% sodium deoxycholate and 0.1% sodium dodecyl sulfate [SDS]). Proteins obtained from CL tissue (100 mg) were electrophoresed using 10% polyacrylamide gel electrophoresis with SDS (SDS-PAGE) and Stain-Free precast gels (Biorad, Hercules, CA, USA). The electrophoresis proteins were transferred to an Immobilon polyvinylidene fluoride (PVDF) membrane (Millipore, Billerica, MA, USA) by means of the wet transfer method. Then, PVDF membranes were blocked with 5% non-fat dry milk in Tris-buffered saline and Tween 20 (TBST) buffer (100 mM Tris–HCl, 0.9% NaCl, and 0.05% Tween 20). The membranes were incubated overnight at 4 °C with the following antibodies: anti-P300 antibody produced in rabbit (1:400), which recognizes the P300 protein with a molecular mass of approximately 264 kDa (Cohesion Biosciences, UK); anti-CREB antibody produced in rabbit (1:130), with a molecular mass of approximately 265 kDa (Cohesion Biosciences, London, UK); SRC-1 antibody (1:200) with a molecular mass of approximately 270 kDa (Sigma, Poznan, Poland); and NCOR-2 antibody, with a molecular mass of approximately 274 kDa (1:400) (Rockville, MD, USA). Western blots for each coregulator were performed with *n* = 5. The membranes were washed three times for 10 min each with TBST buffer and subsequently treated with horseradish peroxidase (HRP)-conjugated anti-rabbit IgG secondary antibody (1:50,000). Immunoreactive bands were detected using the Clarity Western ECL Blotting Substrate (Biorad, Hercules, CA, USA). Band intensity was measured using ImageLab analysis software (Biorad, Hercules, CA, USA) and normalized to total protein in each lane [16].

### 2.7. HAT and HDAC Activities

Slices from corpora lutea from days 2–5, 6–10, 11–16, and 17–20 served as the material for nuclear protein isolation, using the Nuclear/Cytosol Fractionation Kit (Biovision, Milpitas, CA, USA). Isolated proteins were used to determine the activity of HAT and HDAC, using the HAT Activity Colorimetric Assay Kit (Biovision, Milpitas, CA, USA) and the HDAC Activity Colorimetric Assay Kit (Biovision, Milpitas, CA, USA), respectively, according to the manufacturer’s instructions. The materials for the study were the previously isolated nuclear protein extracts.

### 2.8. Data Analysis

Hormone concentration values, real-time PCR, and Western blots are presented as the mean ± SEM; they were compared by one-way analysis of variance (ANOVA) followed by the Tukey test. We analyzed the relative mRNA quantification data with the Real-time PCR Miner algorithm [17]. Data obtained from real-time PCR were normalized to TBP to obtain arbitrary units for the relative amount of the PCR product. Data obtained in Western blot were normalized to total protein in each lane using Stain-Free technology and chemiluminescent protein detection [16]. We performed all calculations using the Graph Pad Prism 8.0 software package (GraphPad Software, Inc., San Diego, CA, USA).

## 3. Results

### 3.1. Immunohistochemistry

**Figure 1:**
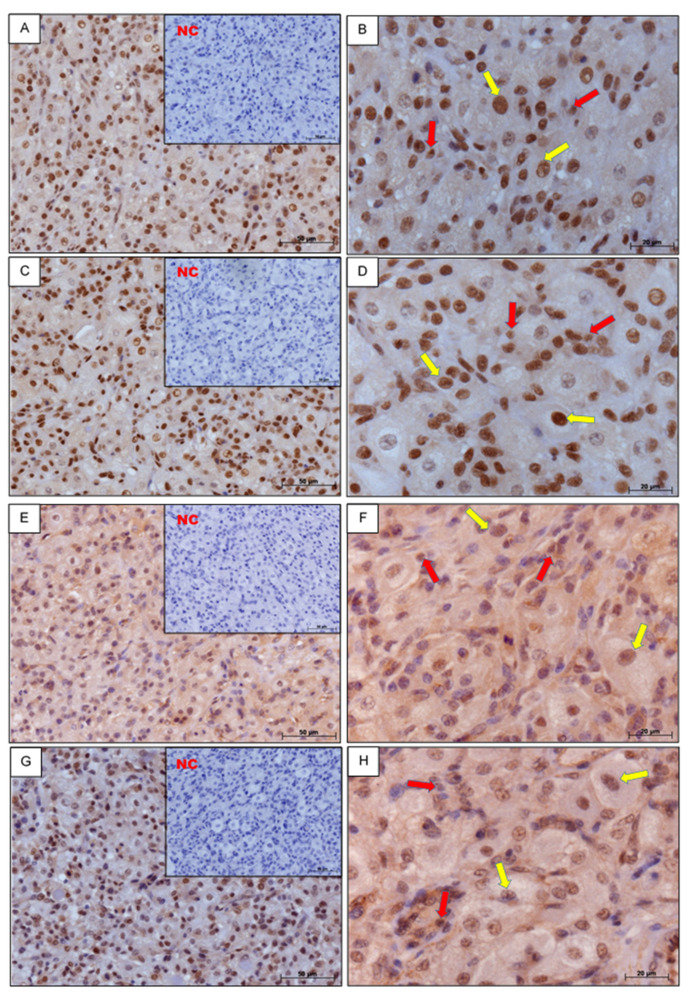
The cellular localization of coactivators histone acetyltransferase p300 (P300) (**A**,**B**), cAMP response element-binding protein (CREB) (**C**,**D**), steroid receptor coactivator-1 (SRC-1) (**E**,**F**), and corepressor nuclear receptor corepressor-2 (NCOR-2) (**G**,**H**) in bovine CL on days 6–10 of the estrous cycle. Negative control (NC) was performed without primary antibodies (smaller box inside left pictures). Yellow arrows denote large luteal cells; red arrows denote small luteal cells. Scale bars: 50 μm and 20 μm.

Positive immunostaining for coactivators P300, CREB, and SRC–1 and corepressor NCOR-2 in CL slices on days 6–10 of the estrous cycle was observed in the nuclei of small and large luteal cells. Negative control sections for all investigated proteins were consistently free of stain (Figure 1).

### 3.2. Progesterone Concentration

**Figure 2:**
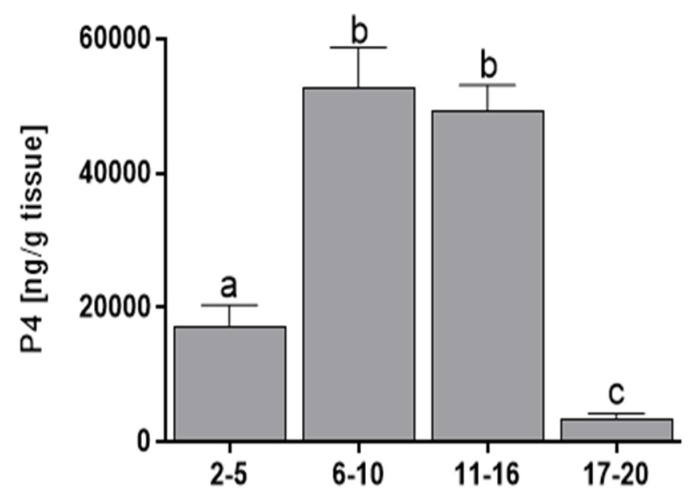
The mean (± SEM) progesterone (P4) concentrations in bovine corpora lutea collected on days 2–5, 6–10, 11–16, and 17–20 (*n* = 5 per stage) of the estrous cycle. Values with different superscripts are different (*p* < 0.05).

The luteal concentration of P4 was higher during days 6–16 of the estrous cycle than during days 2–5 (*p* < 0.01) or 17–20 (*p* < 0.001) (Figure 2).

### 3.3. Expression of Coregulators mRNA in the CL

**Figure 3:**
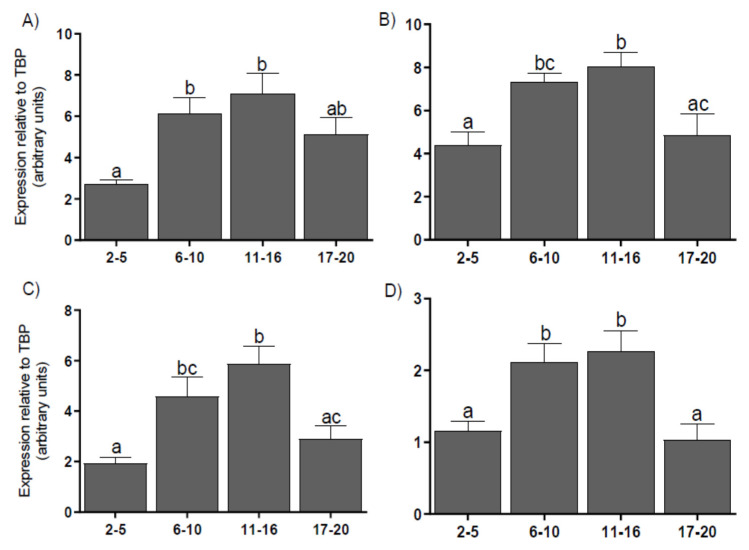
The mean (± SEM) mRNA expression for histone acetyltransferase p300 (P300) (**A**), cAMP response element-binding protein (CREB) (**B**), steroid receptor coactivator-1 (SRC–1) (**C**), and nuclear receptor corepressor-2 (NCOR–2) (**D**) levels in bovine corpora lutea, collected on days 2–5, 6–10, 11–16, and 17–20 (*n* = 5 per stage) of the estrous cycle. Values with different superscripts are different (*p* < 0.05).

The mRNA expression levels for coregulators *P300* (Figure 3A), *CREB* (Figure 3B), *SRC-1* (Figure 3C), and *NCOR-2* (Figure 3D) in the CL were higher on days 6–16 than on days 2–5 (*p* < 0.05) or 17–20 (*p* < 0.05–0.01). We found a correlation between P4 levels and the mRNA expression levels of coactivators *P300*, *CREB*, *SRC-1*, and corepressor *NCOR-2*, as well as between mRNA for *NCOR-2* corepressor and the mRNA for each coactivators (Table 2).

### 3.4. Protein Level of Coregulators in the CL

**Figure 4:**
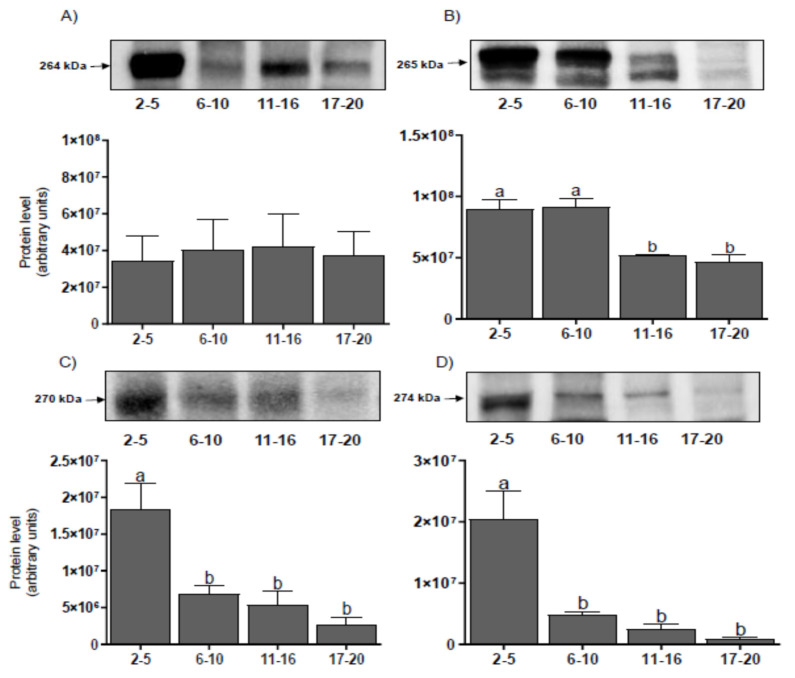
The mean (± SEM) protein levels for histone acetyltransferase p300 (P300) (**A**), cAMP response element-binding protein (*CREB*) (**B**), steroid receptor coactivator-1 (*SRC–1)* (**C**), and nuclear receptor corepressor-2 (*NCOR–2)* (**D**) in bovine corpora lutea, collected on days 2–5, 6–10, 11–16, and 17–20 (*n* = 5 per stage) of the estrous cycle. The upper panel shows a representative Western blot for each of the tested proteins. Values with different superscripts are different (*p* < 0.05).

The protein levels for P300 (Figure 4A) did not show statistical differences during the estrous cycle. CREB protein levels were higher on days 2–10 than on days 11–20 (*p* < 0.001) (Figure 4B). Protein levels for SRC–1 (Figure 4C) and NCOR–2 (Figure 4D) were the highest on days 2–5 and decreased on days 6–20 (*p* < 0.001). A correlation has been found between the protein level for NCOR-2 corepressor and the protein levels for CREB and SRC-1 (Table 2).

### 3.5. Histone Acetyltransferase and Histone Deacetylase Activities

**Figure 5:**
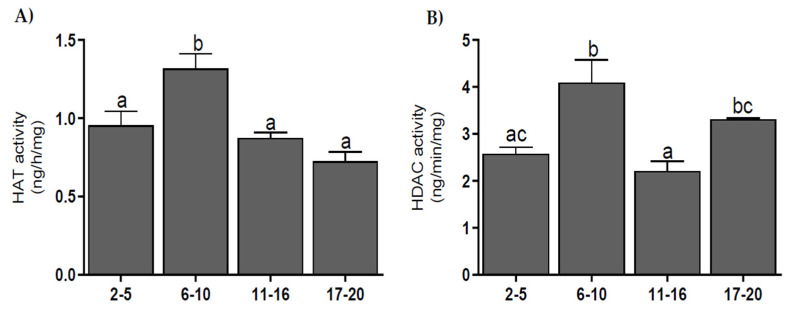
Histone acetyltransferase (HAT) activity (**A**), and histone deacetylase (HDAC) activity (**B**) in bovine corpora lutea, collected on days 2–5, 6–10, 11–16, and 17–20 (*n* = 5 per stage) of the estrous cycle. Values with different superscripts are different (*p* < 0.05).

The activity of HAT was higher on 6–10 d than on other days of the estrous cycle (*p* < 0.01–0.001) (Figure 5A). The activity of HDAC was highest on days 6–10 (*p* < 0.001) and decreased on days 11–16 (*p* < 0.001) and increased again on days 17–20 (*p* < 0.05) (Figure 5B). The activities of HAT and HDAC weakly correlated during the estrous cycle (*r* = 0.51; *p* < 0.05).

## 4. Discussion

Immunohistochemical analysis shows the localization of proteins for coactivators P300, CREB, and SRC-1, and corepressor NCOR-2 in the nuclei of small and large luteal cells in the CL. Earlier studies have also shown the presence of the PGR nuclear receptor in the same types of cells in bovine CL [18]. It should be noted that both PGR and coregulators occur in the same luteal cells. This could indicate that they can interact with each other and thus mediate P4 in the CL.

We found a positive correlation between the mRNA levels of all investigated coregulators and the P4 concentration in CL during the cycle. Moreover, our earlier studies showed the levels of mRNA and proteins for the PGRA and PGRB isoforms of P4 receptor were the highest at the beginning of the estrous cycle and decreased in following estrous stages [19]. We also observed the positive correlation between P4 level and expression of mRNA and protein of the P300/CBP-associated factor (PCAF) coactivator and the NCOR1 corepressor in CL [10]. This may indicate that the mRNA expression of the coregulators may depend on the level of P4. It should be noted that coregulators by binding to the PGR receptor cause its activation/inhibition, subsequently regulating the transcription of PGR connected genes [20]. Therefore, the correlation between the mRNA of the studied coactivators and the P4 level may suggest that coactivators modify the effects of P4 on target genes, and hence, it is possible that P4 is a factor that can regulate the expression of coregulators.

The coactivators SRC-1, P300, and CREB are part of the protein complex that binds to the PGR receptor dimer after they connect to a hormone response element in the activated gene promoter [21]. Therefore, it seems that they can equally participate in the gene activation process by the PGR receptor. This is confirmed by the fact that our study shows that these three coactivators have similar levels of mRNA and proteins during the entire estrous cycle in the CL.

The obtained results indicate that the highest level of mRNA expression for the tested coactivators in CL occurred in days 6–16 of the estrous cycle, with the lowest levels at the beginning and the end of the cycle. We observed similar results in our previous studies showing changes in mRNA expression of the PCAF in cattle CL during the estrous cycle [10]. Moreover, we also showed that on days 6–10 of the cycle, P4 regulates its own synthesis by stimulating the activity of 3-β-hydroxysteroid dehydrogenase/D-5-4 isomerase (3b-HSD) [22]. At the same time, P4 also increases mRNA and protein expression for 3b-HSD StAR and cytochrome P450scc [23], but it has no effect on PGR mRNA levels on days 6–16 of the estrous cycle [24,25]. Therefore, it is possible that P4, by regulating the expression levels of coactivators, may also support its own synthesis and thus indirectly affect the regulation of CL function.

The peak of protein expression for each tested coregulator occurs before the peak of their mRNA expression. We found the highest level for all proteins at the beginning of the cycle. It is possible that the coregulator protein present in CL at the beginning of the cycle is derived from mRNA expressed in follicular cells [26,27]. This possibility is confirmed by the data indicating the presence of PGR and their coregulators in granulosa, theca, and stroma cells [26,27]. Then, the high level of protein in CL at the beginning of the cycle may be the result of mRNA translation that was in the above-mentioned cell types before the ovulation. Meanwhile, a high mRNA expression of coregulators in the middle phase of the cycle may be a part of the regulatory mechanism for potential pregnancy and can be translated to the appropriate protein in CL during the early pregnancy. This supports the increase in P4 level in bovine CL during weeks 3–12 of pregnancy and the increased expression of mRNA and proteins for both PGR isoforms, as was shown earlier [19,28]. Additionally, this effect may be also due to the existence of various post-transcriptional and post-translational mechanisms. The proteins also differ significantly in their half-life, which affects their levels in Western blot measurements [29].

Coactivators also possess epigenetic activity, showing the action of HAT, which acetylates histone proteins and enhances the activity of various genes [30]. The results show that HAT activity is highest on days 6–10 of the estrous cycle. This period is characterized by an increased intensity in angiogenic processes. Research by Zecchin et al. [31], performed in umbilical vein endothelial cells, has shown that the acetylation of vascular endothelial growth factor receptor 2 (VEGFR2) significantly alters the kinetics of receptor phosphorylation after ligand binding, allowing receptor phosphorylation and intracellular signaling upon prolonged stimulation with vascular endothelial growth factor (VEGF). Similar research on human primary aortic endothelial cells (HAEC) has shown that the acetylation of transcription factor E2F1 (E2F1) induces expression of the VEGF receptor [32]. Therefore, high HAT enzyme activity on days 6–10 in cattle may contribute to the formation of new vessels in the CL.

On days 17–20 of the estrous cycle, the levels of the investigated coactivators’ mRNA and proteins were decreased, as well as the P4 level. A lack of fertilization directs luteal cells to the apoptosis pathway [33]. There are significant changes in cell nuclei structures, resulting in chromatin condensation and the enhancement of endonucleases that cause DNA fragmentation [1]. This also induces a decrease in P4 levels and the mRNA and protein expression of individual PGR isoforms [19].

Corepressors exert the opposite effect as coactivators, as shown by NCOR-2 in our research. By interacting with the nuclear receptor, corepressors inhibit the transcription of a given gene [8]. Our results show positive correlations between NCOR-2 corepressor mRNA or protein levels, and between the mRNA or protein levels for each coactivator. This may be due to the similar structure of binding sites for coactivators and corepressors. Coactivators interact with the nuclear receptor by connecting to the LBD (ligand-binding domain) of the receptor through a nuclear receptor box (NR box) containing three leucine and two non-specific amino acids in the sequence LXXLL [34,35]. In contrast, corepressors have additional external amino acids surrounding this motif (I/L)XX(I/V)I [36]. In the absence of ligand availability or the presence of the receptor antagonist, the conformation of the coregulator binding site may change, which leads to the formation of a site corresponding to the binding of corepressors. It has been also found that mutations induced within the binding motif of coactivators in humans result in the inhibition of the attachment of both coactivators and corepressors to the thyroid receptor (TRb) [34,35,37]. Therefore, similar levels of mRNA or protein expression for the tested coactivators and corepressors may indicate that their attachment to the receptor may occur in a competitive manner.

Corepressors also show HDAC activity. By deacetylating histone proteins, they increase the density of chromatin and block the transcription of a given gene [30]. We have shown a very weak correlation (*r* = 0.51) between HAT and HDAC activity in the bovine estrous cycle. The balance between acetylation and deacetylation processes plays an important role in maintaining cell homeostasis [38]. Any disturbance of this balance between HAT and HDAC activity may result in uncontrolled activity or the inhibition of many key genes [39]. Therefore, the similar activity and expression of HAT and HDAC obtained in our experiments is an element of maintaining the balance between cellular processes needed for proper functioning.

The results of our research also show an increase in HDAC activity at the end of the estrous cycle. At this time, the P4 level decreases, while PGF2α secretion increases. This prostaglandin plays a key role in luteolysis in the CL, inhibiting the expression of StAR, which decreases the intracellular transport of cholesterol and the production of steroids, including P4 [40]. PGF2α in rat luteal cells has been found to attach the Yin Yang 1 repressor protein (YY1) to the StAR promoter and increase HDAC activity on this promoter, thereby preventing StAR activation and P4 synthesis [41], while increased HDAC activity at the end of the cycle may support the luteolytic activity of PGF2α.

## 5. Conclusions

mRNA and protein levels for coregulators change with P4 levels, suggesting the role of this hormone in regulating their expression and activities. On the other hand, a similar level of expression between these coregulators and corepressors in the CL may indicate that their attachment to the PGR receptor possibly occurs in a competitive manner. These similar expression levels also form an element of homeostasis in the cell, balancing the expression and silencing of genes significant for cellular functioning.

## Figures and Tables

**Table 1:** Forward and reverse primer sequences used in real-time PCR. Every primer set was designed according to the accession number in the Nucleotide NCBI database. F—forward and R—reverse. Abbreviations: F—forward; R—reverse.

Gen Name	Primers	GenBank Accession Number	Amplicon Length
***P300***	F: CCATGAGCAACATGAGTGCTAGT R: CATTGTCACTCATCAGTGGGTTTT	XM_027540695.1	129
***CREB***	F: TGAAGTGAAGGTCGAAGCTAAAGA R: GTACAGAGCTTCCAGGGTTGACAT	XM_024984694.1	147
***SRC-1***	F: CCCAGGCAGACGCTAAACAG R: TCAAGATAGCTTGCCGATTTTG	XM_028514416.1	114
***NCOR-2***	F: AGCCCTCGAGGCAAAAGC R: CATGCGGAGAGGCCTTGA	XM_024977670.1	177
***TBP***	F: CAGAGAGCTCCGGGATCGT R: ACACCATCTTCCCAGAACTGAATAT	NM_001075742	194

**Table 2:** Coefficients of correlation for the progesterone (P4), histone acetyltransferase (HAT) activity, and histone deacetylase (HDAC) activity, mRNA of the corepressor to mRNA and protein level of the corepressor to the protein level of coactivators of histone acetyltransferase p300 (P300), cAMP response element-binding protein (CREB), steroid receptor coactivator-1 (SRC-1), and nuclear receptor corepressor-2 (NCOR-2) and correlation for HAT and HDAC activities in cow corpus luteum (CL) during the estrous cycle. Abbreviation: ns, not significantly different.

	P300	CREB	SRC-1	NCOR-2
**P4–mRNA**	*r* = 0.59 *p* < 0.001	*r* = 0.56 *p* < 0.05	*r* = 0.76 *p* < 0.0001	*r* = 0.79 *p* < 0.0001
**P4–protein**	ns	ns	ns	ns
**mRNA corepressor** **mRNA coactivators**	*r* = 0.79 *p* < 0.0001	*r* = 0.75 *p* < 0.0001	*r* = 0.89 *p* < 0.0001	−
**Protein corepressor** **Protein coactivator**	ns	*r* = 0.79 *p* < 0.01	ns	−
**HAT–HDAC**	*r* = 0.51 *p* < 0.05			

“ns” means not specified and was included into description.
